# Metabolic interactions: how gut microbial metabolites influence colorectal cancer

**DOI:** 10.3389/fmicb.2025.1611698

**Published:** 2025-07-23

**Authors:** Qinhan Cao, Meiju Yang, Min Chen

**Affiliations:** ^1^Hospital of Chengdu University of Traditional Chinese Medicine, Chengdu, China; ^2^School of Clinical Medicine, Chengdu University of Traditional Chinese Medicine, Chengdu, China

**Keywords:** colorectal cancer, gut microbiota, microbial metabolites, biomarkers, therapeutic strategies

## Abstract

Colorectal cancer (CRC) is a growing public health concern due to its rising incidence and high rate of cancer-associated deaths. Emerging evidence suggests that gut microbiota and their metabolites are critically involved in the initiation and advancement of CRC. These metabolites, which originate from the breakdown of nutrients from food and host-related substances through microbial activity in the gut, can profoundly influence tumor formation. In addition to well-studied compounds such as short-chain fatty acids (SCFAs), bile acids (BAs), tryptophan metabolites, and polyamines, this review highlights emerging metabolites—including hydrogen sulfide (H₂S) and formate—that have recently drawn attention for their roles in colorectal carcinogenesis. We also incorporate recent mechanistic insights, such as butyrate-induced ferroptosis and H_2_S-mediated protein persulfidation, to illustrate how microbial metabolites influence cancer cell metabolism. Moreover, the potential of microbial metabolites as biomarkers for early diagnosis and prognosis of CRC is discussed. Therapeutic strategies targeting microbial metabolites—such as dietary modulation, combination therapies, fecal microbiota transplantation (FMT), and phage therapy—are also reviewed. By providing a comprehensive and up-to-date overview of microbial metabolic networks associated with CRC, this review underscores the critical functions of gut microbial metabolites in tumorigenesis, offering novel insights into their utility as diagnostic and prognostic biomarkers, as well as promising therapeutic targets.

## 1 Introduction

Colorectal cancer (CRC) is one of the most frequently identified cancers and ranks second in cancer-related mortality across genders, making it a significant public health concern (Song and Chan, 2019). Although the overall incidence of CRC has declined in recent years, particularly among older adults, an alarming rise in both incidence and mortality has been observed in younger populations. Most CRC cases are sporadic and progress from adenoma to carcinoma through multiple genetic and epigenetic alterations (Song et al., 2020). Among these alterations, gut microbiota dysbiosis, which characterized by disrupted microbial community, has attracted more and more attention in the field of CRC research (Zou et al., 2018; Chang et al., 2025; Sameni et al., 2025). This dysbiosis in the gut microbiota correlates with various disturbances, including reduced microbial diversity, depletion of beneficial bacteria, and overgrowth of pathogenic microorganisms, all of which contribute to colorectal tumorigenesis (Hanus et al., 2021; Feng et al., 2023). Recent advances in metabolomics and metagenomics technologies have revealed a complex relationship between microbial metabolites and CRC progression, particularly their impact on the tumor microenvironment (Gao et al., 2022; John Kenneth et al., 2023). Notably, specific metabolites such as propionate exhibit protective, anti-tumorigenic functions, while others like TMAO is associated with pro-carcinogenic activities (Wang and Hu, 2025). Beyond their mechanistic roles, microbial metabolites are gaining attention as promising non-invasive biomarkers for CRC screening and prognosis. They can be detected in stool, blood, urine, and even exhaled breath, providing insight into tumor-associated metabolic shifts (Alustiza et al., 2023). Furthermore, manipulating microbial metabolite profiles through dietary intervention, chemotherapy, immunotherapy, fecal microbiota transplantation (FMT), or phage therapy has demonstrated potential to enhance therapeutic outcomes and reduce treatment-related toxicity (Li S. et al., 2024; Abdeen et al., 2025).

This review provides a comprehensive synthesis of current knowledge on how gut microbial metabolites influence CRC onset and progression, highlights their diagnostic and prognostic value, and discusses emerging strategies targeting these metabolites for therapeutic benefit. By integrating metabolic, microbial, and clinical perspectives, this review aims to provide a comprehensive understanding of microbial metabolic networks involved in CRC and to explore emerging therapeutic strategies that target microbial metabolites.

## 2 Key gut microbial metabolites and their impact on CRC development

### 2.1 Short-chain fatty acids

Short-chain fatty acids (SCFAs), notably butyrate, acetate, and propionate, are key metabolites generated during the microbial breakdown of dietary fiber in the colon. These metabolites are essential for sustaining gut homeostasis and have been extensively recognized for their protective influence on the gastrointestinal tract, especially in reducing the risk of CRC (Wang et al., 2019; Gomes et al., 2020).

Butyrate, in particular, is a key fatty acid known for its strong anti-inflammatory and anticancer effects. It serves as the primary metabolic fuel for colon epithelial cells and supports epithelial maturation while preserving cellular integrity (Zeng et al., 2020). A key mechanism of butyrate is the inhibition of histone deacetylases (HDACs), which activates the tricarboxylic acid (TCA) cycle, reverses the Warburg effect, and induces apoptosis in CRC cells. In addition, SCFAs have been found to modulate the Wnt/β-catenin signaling pathway, a key pathway in CRC development and progression. In mouse models, butyrate-generating microbes like *Clostridium butyricum* suppress intestinal tumor formation by inhibiting the Wnt/β-catenin signaling pathway, which highlights the promising application of SCFAs such as butyrate in CRC therapy. Butyrate also protects the intestinal mucosal barrier by activating the AMPK signaling pathway, inducing MUC2 secretion, and maintaining tight junctions (Chen and Vitetta, 2021). In addition, butyrate modulates antitumor immunity by affecting CD8 + T cell activity (He et al., 2021), which plays a significant role in immune surveillance against CRC. Furthermore, a recent study suggests that SCFAs can inhibit the development of CRC by regulating ferroptosis and immune responses (Cui W. et al., 2024). Butyrate, in particular, promotes ferroptosis by downregulating ferroptosis-related genes such as GPX4 and SLC7A11, enhancing the sensitivity of CRC cells to ferroptosis and inhibiting tumor growth.

Acetate, also known as acetic acid, is the most abundant SCFA in the colon, accounting for approximately 60%. It is primarily produced by *Bacteroides* bacteria in the gut through the fermentation of dietary fibers (Bishehsari et al., 2018; Zeng et al., 2020). Acetate can promote apoptosis in CRC cells by inducing changes in lysosomal membrane permeability, which leads to the release of lysosomal protease cathepsin D (CatD) into the cytoplasm, potentially contributing to its anti-cancer effects (Marques et al., 2013). In high-risk CRC populations, acetate concentrations are significantly lower, suggesting that acetate may have a potential protective role in the prevention of CRC. According to a systematic meta-analysis, acetate concentrations were significantly lower in high-risk individuals compared to healthy controls, with a standardized mean difference (SMD) of 2.02 (95% CI: 0.31–3.74, *p* = 0.02). Additionally, in the CRC incidence analysis, the concentration of acetate was significantly higher in healthy controls than in CRC patients, with a SMD of 0.61 (95% CI: 0.09–1.13, *p* = 0.02) (Alvandi et al., 2022).

Propionate, also primarily produced by *Bacteroides*, has been shown to exert potent anti-cancer effects in CRC. These protective effects are primarily mediated through the enhancement of intestinal barrier integrity and the modulation of immune responses. Specifically, propionate promotes the differentiation of regulatory T cells (Tregs) and suppresses the production of pro-inflammatory cytokines, thereby reducing intestinal inflammation and inhibiting CRC development (Wang et al., 2019). In addition, studies have shown that propionic acid promotes apoptosis of CRC cells through multiple mechanisms, including mitochondrial dysfunction, release of apoptosis-inducing factors, activation of caspase-3, down-regulation of PRMT1, and up-regulation of TNFAIP1 (Ryu et al., 2019; Gomes et al., 2020; Ryu et al., 2022).

Formate is another type of SCFA, but compared to other SCFAs, its concentration in the gut is relatively low. It is often considered a secondary intermediate in metabolic processes, but recent studies have revealed its important role in promoting CRC development (El Tekle and Garrett, 2023; Kulecka et al., 2024). Formic acid promotes CRC through a variety of mechanisms, the most obvious of which is through its induction of oxidative stress and DNA injury. Excessive accumulation of formic acid in the colorectal environment can promote reactive oxygen species (ROS) generation via the Fenton reaction, leading to DNA damage, genetic mutations in epithelial cells, and ultimately facilitating colorectal carcinogenesis (Ternes et al., 2022b). In addition, formic acid has been shown to activate several carcinogenic pathways, one of which is the Wnt/β-catenin signaling pathway. Aberrant Wnt signaling, a hallmark of CRC, governs epithelial homeostasis and cellular proliferation (Yin et al., 2023). The ability of formic acid to disrupt this pathway may further exacerbate tumorigenesis. The second is the NF-κB signaling pathway, which is activated by formic acid to trigger immune-related reactions, with chronic inflammation serving as a critical factor in driving CRC progression (Cao et al., 2024). Elevated levels of formic acid have been shown to disrupt the intestinal barrier by downregulating tight junction proteins, leading to increased intestinal permeability (Yu et al., 2018). This barrier dysfunction permits translocation of microbial products and inflammatory cytokines into deeper tissue layers, exacerbating inflammation and creating a microenvironment conducive to CRC progression. In summary, SCFAs are closely linked to the inhibition of CRC progression through their roles in maintaining intestinal barrier integrity and modulating immune homeostasis. However, under dysbiotic gut conditions, the production or function of SCFAs may be impaired, potentially diminishing their anti-tumorigenic effects and creating a microenvironment conducive to tumor progression (Bultman, 2017).

### 2.2 Bile acid

Bile acid (BAs), primarily synthesized in the liver from cholesterol, are essential for fat digestion (Di Ciaula et al., 2023). Beyond their digestive roles, however, BAs are gaining growing attention for their pivotal involvement in the development and advancement of CRC (Gou et al., 2024; Yang et al., 2025). While SCFAs predominantly exert their effects through anti-inflammatory pathways, BAs primarily activate host receptors to influence tumor growth (Ocvirk and O'Keefe, 2017; Ahmad et al., 2023). Within the colon, BAs are subjected to microbial transformation, converting initial BAs exemplified by chenodeoxycholic acid into secondary bile acids (SBAs), such as lithocholic acid (LCA) as well as deoxycholate acid (DCA) (Zeng et al., 2019). These SBAs exert diverse effects on colon cells, with some promoting cancer and others offering protective effects, depending on their concentration, metabolic pathways, and gut microbiota composition (Ridlon et al., 2016; Jiang et al., 2022). DCA, a well-established carcinogen, has been linked to CRC by inducing inflammation, DNA damage, and oxidative stress (Jia et al., 2018; Ternes et al., 2022a). Clinical trials have shown that elevated levels of DCA are particularly associated with an increased CRC risk in populations consuming high-fat, low-fiber diets (Reddy et al., 1989). The production of DCA is linked to disruptions in the gut microbial community, which promote the growth of microbes that transform primary bile acids (PBAs) into harmful SBAs (Ahmad et al., 2023). Conversely, certain BAs, such as lithocholic acid, have been shown to possess anti-cancer properties. LCA can induce apoptosis and inhibit cancer cell proliferation, thus providing a protective mechanism against CRC (Liu et al., 2022; Kouhzad et al., 2025). This dual role of BAs underscores their complex involvement in CRC progression. While SBAs like DCA promote tumorigenesis through inflammation and oxidative damage, other BAs like LCA counteract these effects by promoting cell death in cancer cells (Yang et al., 2025). The balance between these opposing actions is influenced by factors such as bile acid concentration, microbiome composition, and host genetics (Ahmad et al., 2023).

BAs regulate signaling pathways by binding to cellular receptors (Caliceti et al., 2022). One such receptor is the farnesoid X receptor (FXR), which plays a key role in maintaining bile acid homeostasis and exerts protective effects against inflammation. However, in CRC, FXR expression is often downregulated, and lower FXR levels are associated with poorer clinical outcomes (Sun et al., 2021; Yin et al., 2021). In contrast, another bile acid receptor, the G protein-coupled bile acid receptor 1 (TGR5), has been implicated in promoting inflammation, particularly in the context of inflammatory bowel disease (IBD), a recognized risk factor for CRC. Upregulation of TGR5 in the inflamed intestinal mucosa suggests its contribution to the chronic inflammation that drives CRC development (Liu et al., 2021; Zhang H. et al., 2021). In addition to their receptor-mediated effects, BAs are hydrophobic and can directly interact with cell membranes, disrupting their integrity. These disruptions can induce cell cycle abnormalities, enhance invasiveness, and facilitate tumorigenesis (Payne et al., 2010; Zeng et al., 2019).

### 2.3 Amino metabolites

#### 2.3.1 Tryptophan metabolites

Amino acid metabolites are key mediators of the interplay between the host and microbiota during the development of CRC, with their diverse metabolic products influencing cancer progression through different mechanisms. As a vital amino acid, tryptophan metabolites is widely present in dietary sources including meat, soy products, and seeds (Richard et al., 2009). It undergoes multiple metabolic routes, including kynurenine pathway, serotonin pathway, and indole pathway, predominantly by intestinal microbiota (Yan et al., 2024).

The majority of tryptophan metabolism in the gut occurs via the kynurenine and indole pathways, with the kynurenine pathway accounting for about 90–95% of the metabolism, and indole-related pathways processing around 5% (Wyatt and Greathouse, 2021). Indole and compounds derived from indole, including indole-3-acetic acid (IAA), indole-3-lactic acid, indole-3-propionic acid (IPA), and indole-3-aldehyde, are known for their bioactive properties and play important roles in regulating diverse physiological processes including immune modulation, inflammation, cellular homeostasis within the gut (Li M. et al., 2024; Huang et al., 2025). Indole derivatives, primarily produced through the metabolism of the amino acid tryptophan by gut microbiota, have garnered increasing attention in CRC.

Indole derivatives have been shown to promote intestinal homeostasis by enhancing epithelial barrier function and supporting immune tolerance, which is critical for suppressing chronic inflammation, one of the known risk factors for CRC (Li M. et al., 2024). For example, germ-free mice lacking IPA-producing Clostridium mutants in one animal experiment showed increased intestinal permeability, inflammation, and immune cell infiltration (Krause et al., 2024). In addition, Indole-3-methanol, a derivative of indole present in cruciferous vegetables, has been shown to alleviate colitis in mice by upregulating interleukin-22 (IL-22), a cytokine that strengthens epithelial immunity and modulates intestinal inflammation (Nolan et al., 2021). Moreover, indoles may modulate the gut microbiome by promoting the growth of beneficial bacteria and inhibiting pathogenic microbes (Zhao et al., 2019). A balanced microbiota is essential for maintaining intestinal homeostasis and preventing carcinogenesis. However, the role of indole derivatives in CRC is not a single stimulant (Ye et al., 2022). Other studies have found that although indole-3-acetaldehyde (IAAD) can induce apoptosis of CRC cells at low concentrations, it may promote epithelial-mesenchymal transformation (EMT) and cell invasiveness at high concentrations, potentially accelerating tumor progression (Dai et al., 2024). Interestingly, tryptophan metabolism is often altered in CRC patients, manifested by increased kynuretic production and decreased indole production (Sun et al., 2020). The indole pathway and its metabolites mostly protect the body in the fight against tumors, but kynurenine, as the main metabolite in the kynurenine pathway, is mostly associated with the pathogenesis of CRC (Platten et al., 2019). Kynurenine functions as an endogenous ligand of the aryl hydrocarbon receptor (AhR) and can directly bind to and activate this receptor. In colitis-associated CRC models, increased tumor formation was observed in mice lacking AhR, suggesting a potential tumor-suppressive role for AhR (Safe et al., 2013). However, kynurenine–AhR signaling has also been shown to promote an immunosuppressive tumor microenvironment. Upon AhR activation, kynurenine can inhibit effector T cell function and promote Treg differentiation, thereby facilitating immune evasion and tumor progression (Liu et al., 2024).

Tryptophan can additionally be hydroxylated to produce serotonin (5-HT), a key neurotransmitter (Kuhn and Hasegawa, 2020). While most previous research focused on its role in mood regulation, sleep, and appetite control, recent studies have revealed its close association with the development of CRC. The study has been shown serotonin (5-HT) significantly promotes the clonal formation of CRC stem cells through the receptors HTR1B, HTR1D, and HTR1F, with higher concentrations leading to the formation of larger clones (Zhu et al., 2022). In mouse experiments, treatment with a serotonin synthesis inhibitor resulted in tumor shrinkage and a decreased proportion of CRC stem cells thereby impeding tumor growth (Zhu et al., 2022).

#### 2.3.2 Polyamines

Comprising putrescine, spermine, spermidine, polyamines are low molecular weight amines produced by gut microbiota during their metabolic processes (Li et al., 2020). These polyamines are essential for multiple cellular functions, including cell growth, differentiation, and apoptosis. Dysregulated polyamine metabolism is associated with the advancement of several diseases, particularly CRC (Park and Igarashi, 2013). In CRC, polyamines are essential for promoting cell proliferation, stabilizing nucleic acids, regulating ion channels, and modulating protein functions—processes that collectively contribute to tumor growth (Wu et al., 2025). Studies have found that levels of polyamine metabolites such as cadaverine and putrescine in CRC stool samples are closely associated with increased tumor incidence and poor prognosis (Yang et al., 2019). Overgrowth of specific bacteria such as enterotoxin-producing fragile Bacteroides (ETBF) can lead to excessive production of polyamines (Goodwin et al., 2011), thereby increasing the probability of CRC. Polyamines have been shown to regulate matrix metalloproteinases (MMPs), which play a key role in tumor migration, invasion, and metastasis (Soda, 2011). In addition, polyamines regulate the Wnt/β-catenin signaling pathway through spermidine/spermidine-N (1) -acetyltransferase (SSAT) activity, further promoting tumor proliferation (Wang et al., 2017). Notably, ornithine decarboxylase (ODC), the rate-limiting enzyme in polyamine biosynthesis, regulates cell proliferation and apoptosis, and also promotes tumor angiogenesis through a pro-angiogenic mechanism independent of the conventional VEGF pathway (Holbert et al., 2024). Tumors with high ODC expression show stronger angiogenic ability which is very unfavorable to the control of CRC.

#### 2.3.3 H_2_S

Hydrogen sulfide (H₂S) in the human gut is produced by sulfate-reducing bacteria (SRB) and other gut microbes through the metabolism of sulfur-containing amino acids such as cysteine and taurine. Recent studies have identified a strong association between elevated levels of H₂S-producing bacteria—such as *Bilophila wadsworthia* and *Desulfovibrio*—and an increased risk of CRC, suggesting a close link between H₂S and CRC pathogenesis (Ai et al., 2019). Firstly, H₂S is essential for modulating the bioenergetic processes of CRC cells. H₂S exhibits a biphasic effect in cancer biology: at low concentrations, it enhances mitochondrial function and promotes glycolysis, thereby supporting cancer cell proliferation, whereas at high concentrations, it inhibits proliferation. For example, the slow-releasing H₂S donor GYY4137 has been shown to stimulate mitochondrial ATP production and enhance glycolysis in CRC cell lines such as HCT116. Low concentrations of GYY4137 (0.3 mM) promote cell proliferation by enhancing bioenergetics, whereas higher concentrations (1 mM) inhibit cancer cell growth (Untereiner et al., 2017). Secondly, H₂S can promote tumor progression through persulfidation—a post-translational modification in which a sulfur atom is added to the cysteine residue of a protein, converting –SH to –SSH. This modification enhances the activity of several pro-tumor signaling pathways, thereby promoting cancer cell proliferation, inhibiting apoptosis, and facilitating tumor growth. For instance, H₂S-mediated persulfidation activates the NF-κB pathway, which not only sustains chronic inflammation but also supports tumor angiogenesis and progression. At the same time, H₂S also activates the downstream mTOR signaling pathway by promoting the persulfidation and activation of AKT, which drives the metabolic reprogramming and rapid division of tumor cells (Wang et al., 2021). In addition, in CRC cells, H₂S regulates Sp1 transcription factors and promotes the activation of ATP citrate lyase (ACLY) genes, further triggering a series of biological effects that are conducive to tumor cell growth and metabolic reprogramming (Ascenção et al., 2021).

### 2.4 Trimethylamine n-oxide

Trimethylamine n-oxide (TMAO) is another compound formed through the combined activities of gut microbiota and liver enzymes. Foods rich in compounds containing trimethylamine groups, such as choline, betaine, and carnitine, found in fish, red meat, and poultry, serve as precursors for TMAO production (Liu and Dai, 2020). Although the majority of research has focused on the relationship between TMAO and cardiovascular diseases, recent studies suggest that TMAO may also play a role in the progression of CRC by promoting cell proliferation and angiogenesis. *In vitro* experiments have demonstrated that TMAO induces proliferation of CRC HCT-116 cells and increases the secretion of vascular endothelial growth factor A (VEGFA). *In vivo*, mice subjected to a long-term high-choline diet exhibited elevated circulating TMAO levels, increased tumor volume, enhanced angiogenesis, and upregulated expression of VEGFA and CD31 (Yang S. et al., 2022). These findings indicate a potential association between TMAO and CRC progression. Furthermore, a prospective study involving 761 CRC cases revealed that TMAO and its precursors are positively associated with the risk of distal colon cancer, while no significant association was found with the overall risk of rectal cancer (Byrd et al., 2024). This suggests that TMAO may influence CRC risk differently depending on the tumor’s anatomical location. However, the precise mechanisms underlying the relationship between TMAO and CRC remain unclear. Further research is necessary to elucidate the pathophysiological mechanisms and causal relationships between TMAO and CRC development.

## 3 Microbial metabolites as biomarkers for CRC diagnosis and prognosis

CRC is a highly heterogeneous disease, posing significant challenges for early detection and accurate prognostication in clinical oncology. Traditional diagnostic methods have many disadvantages, such as colonoscopy and histopathology are invasive and expensive, while biomarkers like carcinoembryonic antigen (CEA) and microsatellite instability (MSI) exhibit limitations in sensitivity and specificity, often leading to false-positive results. Therefore, as a valuable biomarker, the detection of microbial metabolites associated with colorectal tumors came into being. Microbial metabolic profiling provides a non-invasive method for the diagnosis and prognosis of CRC (Feng et al., 2023), which is expected to improve diagnostic accuracy and patient survival rates.

### 3.1 Identification of metabolites as diagnostic markers

**Figure 1:**
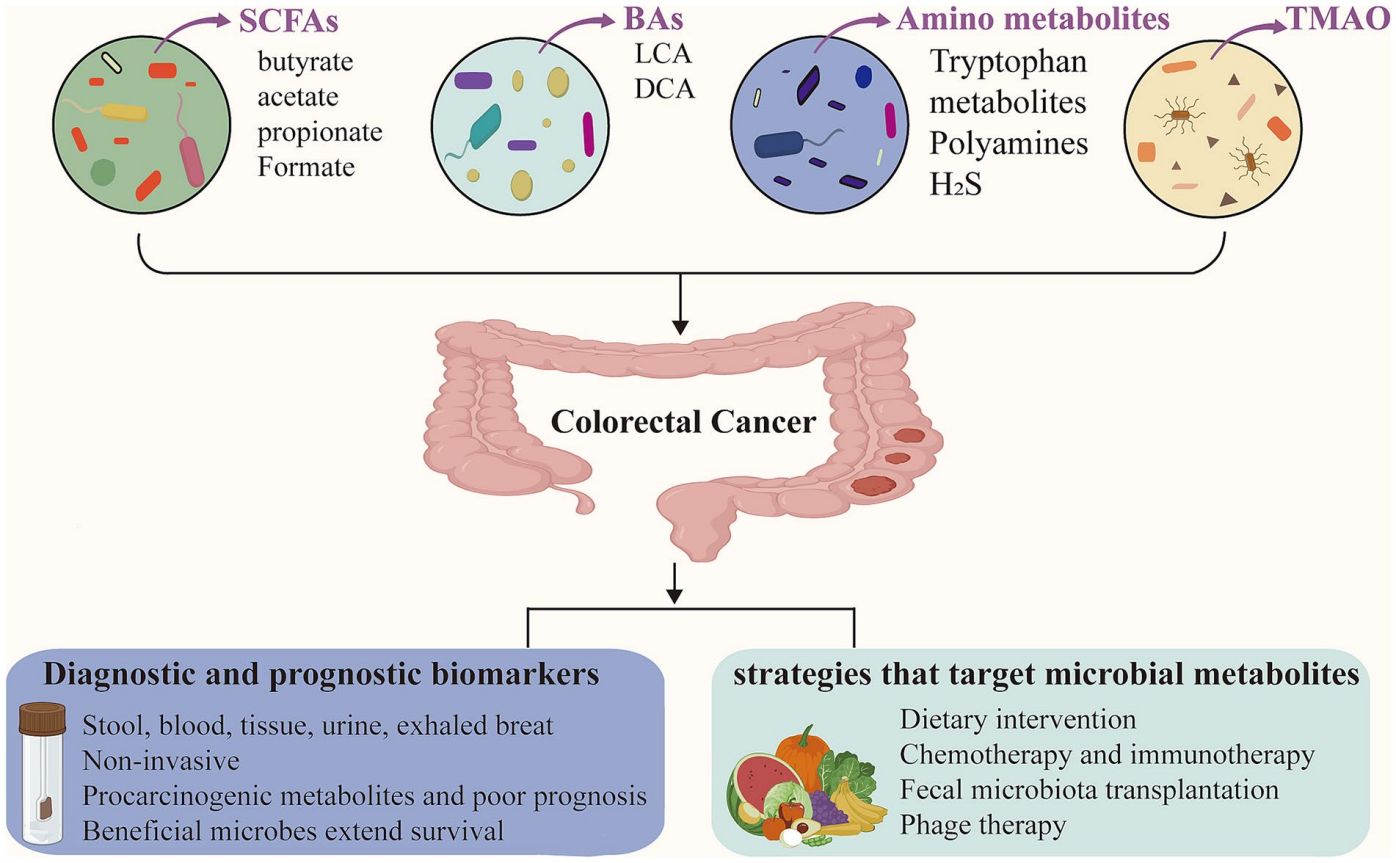
Gut microbial metabolites in colorectal cancer. Microbial metabolites, such as short-chain fatty acids (SCFAs), bile acids (BAs), amino acid derivatives, and trimethylamine-N-oxide (TMAO), play important roles in the modulation of colorectal cancer (CRC). These metabolites can serve as non-invasive diagnostic and prognostic biomarkers, detectable in stool, blood, tissue, urine, and exhaled breath. Furthermore, microbial metabolites offer emerging insights for guiding CRC treatment strategies.

Identification of microbial metabolites as diagnostic biomarkers for CRC has attracted considerable scientific interest. These gut microbial metabolites are detectable in various biological specimens, including stool, blood, tissue, urine, and exhaled breath (van Vorstenbosch et al., 2022). Notably, CRC patients exhibit dynamic shifts in microbial metabolite profiles, reflecting the complex interplay between the gut microbiome and tumor progression. Figure 1 provides an overview of the sample types and representative microbial metabolites used for CRC diagnosis.

Stool samples represent one of the most accessible and informative sources for microbial metabolite analysis, providing a direct reflection of gut microbial activity. A study involving 441 participants found that the fecal microbiota diversity of young CRC patients was significantly lower than that of healthy controls (*p* = 0.0074), and the abundance of butyrate-producing bacteria (such as *Faecalibacterium prausnitzii* and *Eubacterium rectale*) was significantly reduced (Kong et al., 2023). Additionally, stool metabolite analysis has revealed significant alterations in SBAs, which are produced by dysbiotic gut bacteria like *Clostridium scindens*. Increased fecal DCA levels correlate with tumorigenic changes in the colon, promoting carcinogenesis via DNA damage and FXR signaling disruption, making it a valuable biomarker for early CRC detection (Ma et al., 2022). Importantly, combining multiple metabolite markers - such as DCA/butyrate and polyamine/SCFA ratios has shown higher discriminatory power compared to single-metabolite measurements alone. Recent studies indicated that the ratio of butyrate to SBAs, such as DCA, offered superior diagnostic specificity in distinguishing early CRC from adenomas (AUC = 0.92). These findings supported the use of multi-metabolite panels to improve diagnostic accuracy and highlight the importance of combinatorial metabolite profiling (Coker et al., 2022).

In addition to stool analysis, other non-invasive methods such as urine and exhaled breath tests have also been employed clinically to detect alterations in gut microbial metabolites in patients with CRC. Certain volatile organic compounds (VOCs) produced by gut microbial metabolism can be excreted through urine and exhaled breath samples. Therefore, analyzing microbial-derived VOCs in urine and exhaled breath offers insights into gut microbiota activity and provides a novel direction for the early diagnosis and screening of CRC. A meta-analysis that included 11 CRC case studies evaluated the diagnostic potential of VOCs in breath and urine samples, identifying 14 microbial-related VOCs in CRC. The pooled sensitivity and specificity were 0.89 and 0.88, respectively, with an AUC of 0.95, indicating the strong diagnostic potential of VOCs for cancer screening (Zhou et al., 2024).

Microbial metabolites can be detected also in blood samples, particularly in serum or plasma, using metabolomics approaches such as liquid chromatography–mass spectrometry (LC–MS/MS). A recent study by Hang et al. (2022) reported that higher plasma levels of the microbial metabolite phenylacetylglutamine (PAG) were associated with a lower risk of CRC precursors, including conventional adenomas and serrated polyps, in women. In line with this finding, another clinical trial analyzed plasma and fecal samples from 1,251 individuals which demonstrated significant differences in the metabolic profiles of patients with CRC and colorectal adenomas (CRA) compared to those in the normal control (NC) group. Notably, plasma from CRC patients exhibited a significant enrichment of *oleic acid* and a marked reduction in *isodeoxycholic acid*. Based on these metabolic changes, the study developed a diagnostic panel comprising 17 plasma metabolites, which achieved AUC values ranging from 0.848 to 0.987, highlighting the strong diagnostic potential of gut microbial metabolites (Sun et al., 2024). Gao’s work identified serum specific gut microbial metabolites, such as *leucylalanine* and *linoleoylglycerol*, which consistently distinguished CRC patients from healthy individuals across multiple cohorts. A diagnostic panel based on these metabolites achieved an AUC of 0.912 for colorectal adenomas (CRA) and 0.994 for CRC, indicating excellent diagnostic performance (Gao et al., 2022). These studies provided a basis for detecting intestinal microbial metabolites in blood samples to predict the progression of CRC. In addition, changing in plasma metabolites serves as an important indicator for determining whether a patient responds to chemotherapy to distinguish responders from non-responders. One study found significant differences in plasma metabolite profiles between chemotherapy responders and non-responders. By analyzing 17 specific plasma metabolites, the study established a diagnostic model with an AUC of 0.908, sensitivity of 86.4% and specificity of 83.3% (Sun et al., 2024).

The collection of tissue biopsy samples, like that of such as blood samples, is an invasive procedure (Cousins et al., 2019). Moreover, tissue biopsies may be limited by the representativeness of the sampling site. Tissue biopsies remain valuable in certain research contexts, particularly when direct analysis of the intratumoral microbiota and associated metabolites is required. Feng’s work compared cancerous lesions and surrounding non-tumorous tissues in CRC patients, along with corresponding stool samples, have revealed distinct microbial and metabolic profiles across these sample types (Feng et al., 2023). Additionally, another study investigated the characteristics of microbial communities and resistomes in biopsy specimens, underscoring the value of tissue samples in elucidating the complexity and dynamics of the gut microbiome (Yuan et al., 2021). When considering gut microbial metabolites as diagnostic biomarkers for CRC, selecting appropriate detection methods based on the clinical context is essential to ensure both clinical relevance and practical feasibility.

### 3.2 Prognostic value of microbial metabolites

Gut microbial metabolites profiling is important to the prognostic assessment of CRC. Alterations in specific gut microbial metabolites may serve as biomarkers for predicting CRC progression and patient outcomes. Several metabolites mentioned above, such as SBAs, TMAO, and H₂S, had been implicated in promoting CRC development by inducing chronic inflammation, DNA damage, and activation of oncogenic signaling pathways (Ajouz et al., 2014; Cheng et al., 2024). Accordingly, elevated levels of these metabolites in CRC patients often associated with poor prognosis. In contrast, specific SCFAs like butyrate are recognized for exerting anti-inflammatory and anti-cancer effects (Feitelson et al., 2023). Higher levels of butyrate in CRC patients are linked to improved prognostic outcomes. Several studies shown that the increased abundance of specific beneficial bacterial taxa and associated metabolites are significantly correlated with prolonged progression-free survival (PFS) and overall survival (OS) in cancer patients. This further supports the prognostic relevance of gut microbial metabolites (Zhu et al., 2024). In addition, Löser et al. demonstrated the potential value of polyamines as prognostic biomarkers for postoperative recurrence and disease progression in CRC patients (Löser et al., 1990). Although serum and urinary concentrations of several polyamines (e.g., total spermidine, acetylputrescine, and N^1^-acetylspermidine) were significantly elevated in CRC patients compared to healthy controls, similar elevations were also observed in certain non-malignant gastrointestinal conditions, limiting their diagnostic specificity. Thus, although polyamines exhibit high sensitivity, their utility as stand-alone diagnostic markers is limited, but their prognostic potential appears more promising. In patients who underwent curative surgery, polyamine levels returned to normal, whereas confirmed tumor recurrence or metastasis was associated with further increases. Moreover, polyamine levels were found to correlate positively with tumor size, although no significant associations were observed with Dukes staging, tumor location, or conventional tumor markers like CEA and CA19-9 (Löser et al., 1990). Overall the results indicate that polyamines may serve as auxiliary prognostic biomarkers for monitoring treatment response and detecting tumor recurrence. In conclusion, gut microbial metabolites show considerable promise as prognostic biomarkers in CRC. Before clinical application, large-scale cohort studies are required, and standardized protocols for sample preparation and metabolomic analysis are essential to ensure objective and accurate assessment of their prognostic utility.

## 4 Novel strategies based on gut microbial metabolites

### 4.1 Dietary intervention

Dietary intervention is crucial for regulating gut microbiota and its metabolites in the management of CRC. The effects of microbiota-targeted dietary treatments on CRC, based on microbial metabolite regulation are summarized in Table 1.

**Table 1:** Summary of dietary patterns targeting colorectal cancer through modulation of microbial metabolites.

No.	Dietary patterns	Relevant metabolites	Disease	Effect	Mechanism	References
1	Fiber-rich diet, plant-based diet	SCFAs	CRC	Beneficial	Stimulate SCFA production, enhancing gut homeostasis and exerting anti-inflammatory effects	Alderweireldt et al. (2022)
2	Fiber-rich diet	SCFAs	CRC	Beneficial	Increase SCFAs production, potentially reducing CRC risk	Hou et al. (2022), John Kenneth et al. (2023)
3	Polyphenol-, polysaccharide and terpenoid-rich diet	SCFAs	CRC	Beneficial	Polyphenols and polysaccharides modulate gut microbiota to increase SCFA levels, contributing to CRC prevention	Bai et al. (2025)
4	Fibrous polysaccharide-rich diet, intermittent fasting	Butyrate	CRC	Beneficial	Increase microbial production of butyrate, supporting host anti-cancer immune pathways	Ahmad et al. (2023)
5	Whole grains and legumes diet	SBAs PBAs	CRC	Beneficial	Increased 3-oxocholic acid, a type of SBA, may support protective regulation in high-risk CRC individuals	Hill et al. (2023)
6	Prebiotic-rich diet	Indole Pipecolic acid	CRC Polyps	Beneficial	Increase pipecolic acid while reducing indole, contributing to anti-tumor metabolic reprogramming	Zhang et al. (2023)
7	High-fiber, low-fat diet	Butyrate SBAs	CRC	Beneficial	Dietary fiber boosts butyrate production, whereas high fat intake increases SBAs	O'Keefe et al. (2015)
8	High-fat and specific nutrient-enriched diet	DCA	CRC	Harmful	Increase production of harmful bile acid metabolites like DCA, promoting CRC development	Cai et al. (2022)
9	High-soluble fiber diet	DCA	CRC	Harmful	Promotes toxic bile acid formation such as DCA, thereby elevating CRC risk	Yang et al. (2024)
10	High-fat diet	DCA SCFAs	CRC	Harmful	Alters gut microbial metabolism, increasing DCA and reducing SCFA levels, ultimately facilitating tumor progression	Yang J. et al. (2022)

CRC, colorectal cancer; SCFAs, short-chain fatty acids; PBAs, primary bile acids; SBAs, secondary bile acids; DCA, deoxycholic acid; IPA, indole-3-propionic acid.

During digestion, gut microbes ferment nutrients such as dietary fiber to produce SCFAs, including butyrate, propionate, and acetate. These metabolites exhibit anti-inflammatory and anti-tumor properties, thereby contributing to intestinal health (John Kenneth et al., 2023). Dietary fiber serves as the essential substrate for the microbial production of SCFAs. A comparative study between African Americans and rural South Africans demonstrated that compared to a diet high in animal protein and fat, a high-fiber, low-fat diet significantly increases butyrate production, inhibits SBAs synthesis (such as DCA), and reduces inflammation and hyperplasia of the colon mucosa, which is associated with the prevention of CRC (O'Keefe et al., 2015). Compared to a diet rich in animal-based proteins and fats, a high-fiber diet particularly the Mediterranean diet, which enhances butyrate production and inhibits histone deacetylase (HDAC) activity through epigenetic mechanisms, thus preventing CRC cells proliferation. Evidence from another study demonstrated a strong association between dietary patterns and risk of distal colon cancer. Diets that promote the enrichment of SCFA-producing bacteria were linked to a reduced risk of distal colon cancer. Specifically, the study showed that for men, the hazard ratio (HR) was 0.65 (95% CI, 0.47–0.89), indicating a significant protective effect. For women, the HR was 0.77 (95% CI, 0.64–0.93), also indicating a significant reduction in risk. This further confirm that a diet rich in fruits, vegetables, low in processed meats, and low in sugary beverages has a protective effect in reducing CRC risk (Lo et al., 2021). Plant-based diets are generally more beneficial than animal-based diets because plant-based diets are rich in unsaturated fatty acids and dietary fiber, which help increase SCFAs production, reduce SBAs production, and lower the risk of CRC (Alderweireldt et al., 2022). Diets rich in polyphenols, polysaccharides, and terpenoids can also indirectly promote the production of SCFAs, thereby potentially reducing the risk of CRC (Bai et al., 2025). In addition, dietary interventions such as dietary fiber polysaccharide intake and intermittent fasting (such as Ramadan fasting) are able to alter the composition of the gut microbiota, promote the growth of beneficial microorganisms, and strengthen the host’s anti-cancer defenses (Ahmad et al., 2023). However, excessive soluble fiber intake may disrupt microbial community composition, favoring the growth of harmful microbes that produce toxic bile acid metabolites, such as DCA, thereby increasing CRC risk (Yang et al., 2024). Additionally, a high-fat diet has been found to reduce the production of beneficial SCFAs while promoting intestinal inflammation through the increased production of harmful bile acid metabolites. This diet also activates key signaling pathways, including Wnt/β-catenin and NF-kB, which contribute to tumor progression. Furthermore, a high-fat diet may elevate the production of harmful metabolites, such as ammonia and nitrites, which promote cancer risk by inducing DNA damage and amplifying inflammation (Yang J. et al., 2022). Studies also suggest that bile acid derivatives such as UDCA may regulate bile acid metabolism and potentially reduce the risk of colon cancer. However, gender differences in its efficacy require further investigation to clarify its therapeutic potential (Cai et al., 2022).

In addition, prebiotic foods can have a positive impact on the treatment of CRC by regulating the intestinal microbiota and its metabolites. The BE GONE trial results demonstrated that after consuming legumes as prebiotic foods, obese patients with a history of CRC or polyps exhibited an increase in blood levels of pipecolic acid, while indole levels decreased (Zhang et al., 2023). The increase in pipecolic acid may reduce the risk of cancer by regulating the inflammatory environment in the intestine and reducing chronic low-grade inflammation, while the decrease in indole levels can help maintain a healthy intestinal environment and reduce the risk of cancer caused by intestinal inflammation. In another study, researchers found that supplementation with a whole grains and legumes diet, such as rice bran and navy beans, had limited effects on SCFAs but led to an increase in 3-oxocholic acid, a type of SBAs, which may support protective regulation in individuals at high risk for CRC (Hill et al., 2023). Additionally, dry legumes as prebiotic foods can promote the growth of gas-producing *Clostridium perfringens*, *Enterococcus faecalis*, and *Bifidobacterium*. Legumes may exert significant therapeutic effects in high-risk populations for CRC by modulating the gut microbiota and its metabolites (Cai et al., 2022). A recent review further reported that gut microbes can convert isoflavones from soy and its products into active metabolites such as equol, which shows strong inhibitory effects on CRC cells. This further confirms the association between soybean consumption and a reduced risk of CRC (Wang et al., 2024).

### 4.2 Chemotherapy

Metabolites produced by the gut microbiota may significantly contribute to CRC chemotherapy by modulating tumor metabolism, enhancing drug sensitivity, and mitigating chemotherapy-induced toxicity (Li S. et al., 2024). Increasing evidence indicates that gut microbiota and their metabolites can substantially influence the pharmacokinetics and therapeutic efficacy of chemotherapeutic agents. For instance, Zimmermann et al. (2019) systematically evaluated the metabolic capacity of 76 human gut bacterial strains against 271 orally administered drugs and found that more than two-thirds of the drugs could be significantly metabolized by at least one strain. These findings highlight the extensive potential of gut microbiota and their metabolites in modulating drug metabolism across various diseases, including CRC. Notably, 5-fluorouracil (5-FU), a key chemotherapeutic for CRC, also shows strong antimicrobial activity against *Fusobacterium nucleatum* (Fn), a bacterium linked to poor prognosis and chemoresistance (LaCourse et al., 2022). This suggests that part of 5-FU’s efficacy may stem from targeting tumor-associated microbes, underscoring the importance of microbiota–drug interactions in CRC treatment.

In this context, specific microbial metabolites have been shown to influence drug efficacy through distinct molecular mechanisms. Among these, butyrate and indole-3-acetic acid (IAA) are well-characterized. Butyrate enhances the efficacy of chemotherapeutic agents via two mechanisms: it inhibits tumor cell glycolysis (the “Warburg effect”), reducing energy supply and sensitizing cells to chemotherapy, and it activates the GPR109a–AKT pathway, inducing cell cycle arrest and apoptosis, thereby increasing chemosensitivity (Geng et al., 2021). IAA, on the other hand, is oxidized by myeloperoxidase (MPO) in tumor-infiltrating neutrophils into cytotoxic derivatives in FOLFIRINOX-treated models. These byproducts elevate ROS levels and downregulate antioxidant enzymes such as GPX3 and GPX7, enhancing oxidative stress (Tintelnot et al., 2023). Simultaneously, IAA inhibits autophagy, further suppressing tumor proliferation. These synergistic effects have been shown to boost the antitumor efficacy of chemotherapy in CRC and other solid tumors (Kwon, 2023). Beyond enhancing cytotoxicity, microbial metabolites also modulate chemoresistance. For instance, butyrate enhances CRC cell sensitivity to irinotecan by promoting apoptosis and reducing chemoresistance-related protein expression (Encarnação et al., 2018). It also facilitates OXA-induced ferroptosis by downregulating SLC7A11 in a c-Fos-dependent manner, overcoming ferroptosis resistance and improving drug efficacy (He et al., 2023). In contrast, some metabolites promote chemoresistance. *Bacteroides vulgatus* boosts nucleotide biosynthesis and DNA repair in tumor cells, enhancing resistance to 5-FU (Teng et al., 2023). H₂S similarly enhances resistance by modulating apoptosis pathways. In CRC cell models, H₂S inhibited chemotherapy-induced apoptosis, contributing to treatment failure. Inhibition of H₂S production, such as through aminooxyacetic acid (AOAA), has been shown to restore chemosensitivity and enhance 5-FU-induced apoptosis (Lin et al., 2023).

In addition to influencing drug efficacy and resistance, gut microbial metabolites also affect chemotherapy-induced toxicities. Butyrate has been shown to promote the differentiation of Tregs, thereby alleviating excessive inflammatory responses induced by chemotherapy. In addition, SCFAs play a protective role in maintaining intestinal mucosal integrity by reducing epithelial cell apoptosis, which helps mitigate chemotherapy-induced gastrointestinal mucosal injury and lowers the incidence of adverse effects such as diarrhea (Al-Qadami et al., 2022). Moreover, butyrate exhibits antioxidant and anti-inflammatory properties that contribute to the attenuation of other systemic toxicities associated with chemotherapy, including cardiotoxicity, by protecting cardiomyocytes from damage (Russo et al., 2019). Therefore, certain natural bioactive compounds that promote the production of SCFAs, such as purple rice bran anthocyanins (PRBA), have shown promising potential as adjunctive therapies in CRC through reshaping the gut microbial composition and boosting antitumor immunity (Chen T. et al., 2022). Building on this, increasing attention has been given to natural polysaccharides used in combination with chemotherapeutic agents (Chen M. et al., 2022). For instance, carboxymethylated pachyman (CMP) and *Albuca bracteata* polysaccharides have demonstrated the ability to stimulate the production of SCFAs as well as to improve gut microbiota composition (Qin et al., 2022). Taken together, the combination of these natural compounds with chemotherapeutic drugs like 5-FU not only helps alleviate chemotherapy-induced gut dysbiosis and toxicity, but may also enhance the overall therapeutic efficacy by improving the intestinal microenvironment and reinforcing immunomodulatory and anti-inflammatory mechanisms. In contrast, some gut microbial metabolites may intensify the systemic toxicity of chemotherapy by interfering with host drug metabolism. For instance, it has been shown that gut microbiota can convert Brivudine into a toxic metabolite, BVU, which is absorbed and disrupts hepatic drug-metabolizing enzymes, thereby increasing the systemic toxicity of agents such as 5-FU. This finding reveals the pivotal role of microbial metabolites in modulating gastrointestinal drug metabolism and shaping therapeutic outcomes and systemic toxicity profiles in distant, sterile sites. Interestingly, certain microbial metabolites may exert dual effects. For example, urolithin A, derived from polyphenol metabolism by the gut microbiota, has been shown to enhance cancer cell sensitivity to chemotherapy-induced stress by regulating oxidative stress and activating mitophagy (D'Amico et al., 2021). At the same time, it downregulates the expression of multiple drug efflux pumps in tumor cells, thereby reversing multidrug resistance (MDR) and increasing the intracellular accumulation and cytotoxicity of chemotherapeutic agents such as cisplatin, paclitaxel, and 5-FU (Ghosh et al., 2022).

### 4.3 Immunotherapy

Treatment strategies for CRC are undergoing a major shift from traditional surgery, chemotherapy, and radiation to immunotherapy. Immunotherapy has become an important part of CRC treatment by enhancing the body’s immune response to identify and eliminate cancer cells. Immune checkpoint blocking (ICB) therapy is an emerging cancer immunotherapy strategy, which alleviates immune suppression and promotes antitumor immunity by blocking the immune checkpoint pathway between tumor cells and immune cells. In CRC, immune checkpoint inhibitors (ICIs) primarily act by blocking the interaction between programmed cell death protein 1 (PD-1) and its ligand PD-L1, and also by inhibiting the function of cytotoxic T lymphocyte-associated antigen 4 (CTLA-4) (Jiao et al., 2020). However, there are many limitations in the clinical application of treatment in patients with CRC. There are significant differences in response to ICB therapy among CRC patients, with studies showing that individuals exhibiting deficient mismatch repair (dMMR) or high levels of microsatellite instability (MSI-H) tend to respond more favorably to ICB treatment, while the efficacy is limited for patients with microsatellite stability (MSS) (Jin and Sinicrope, 2022). In addition, the application of ICB therapy in CRC is also facing the problem of drug resistance. Therefore, immunotherapy studies based on metabolites of gut microbiota provide a new direction to improve the effectiveness of CRC immunotherapy. To further illustrate the regulatory roles of microbial metabolites in immune checkpoint therapy, Table 2 summarizes the current evidence on how representative gut microbial metabolites affect the efficacy and mechanisms of ICIs in CRC.

**Table 2:** Effects of microbial metabolites on immune checkpoint inhibitors.

Microbial metabolites	ICIs	Effect on ICIs efficacy	Mechanism	References
SCFAs	PD-1, PD-L1	Dual	Moderate levels of SCFAs boost anti-tumor immunity by adjusting the immune microenvironment, enhancing the effectiveness of anti-PD-1/PD-L1 treatment. Excessively high SCFA levels may increase immune tolerance	Hou et al. (2022)
SCFAs	PD-1	Reduction	Reduced SCFA production may diminish both the activity and abundance of cytotoxic T cells	Spencer et al. (2021)
Butyrate	CTLA-4	Reduction	Butyrate suppressed CD80/CD86 upregulation on dendritic cells and ICOS expression on T cells, limiting tumor-specific and memory T cell accumulation	Coutzac et al. (2020)
Inosine	PD-1, CTLA-4	Enhancement	Inosine activates the adenosine A2A receptor on T cells and, with IFN-γ and co-stimulation, promotes T cell activation and boosts anti-tumor immunity	Liu et al. (2023)
IPA	PD-1	Enhancement	IPA enhances CD8^+^ T cell stemness by epigenetically activating the Tcf7 super-enhancer, sustaining anti-tumor immunity	Jia et al. (2024)
Gallic acid	PD-1, PD-L1	Enhancement	Gallic acid reprograms Treg cells into Th1-like cells via STAT3–Usp21–FOXP3/PD-L1 signaling, enhancing CD8^+^ T cell-driven anti-tumor immunity	Deng et al. (2022)
EPS-R1	PD-1, PD-L1, CTLA-4	Enhancement	EPS-R1 induce CCR6^+^ CD8^+^ T cells that infiltrate tumors and enhance IFN-γ–mediated anti-tumor immunity	Kawanabe-Matsuda et al. (2022)

SCFAs, short-chain fatty acids; IPA, indole-3-propionic acid; GA, gallic acid; ICIs, immune checkpoint inhibitors; PD-1, programmed cell death protein 1; PD-L1, programmed death-ligand 1; CTLA-4, cytotoxic T-lymphocyte-associated protein 4.

Growing research highlights the significant influence of microbial metabolites in modulating cancer immunotherapy. For instance, SCFAs, in addition to the roles mentioned above, may also be associated with the effects of immune checkpoint inhibitors. One study by Hou et al. found that SCFAs exhibit bidirectional immunoregulatory functions and modulate the therapeutic response to anti-PD-1/PD-L1 immunotherapy. The study indicated SCFAs contribute to strengthening antitumor immune activity via modulation of the tumor immune microenvironment, thus improving the efficacy of anti-PD-1/PD-L1 treatment. However, excessively high levels of SCFAs may lead to increased immune tolerance (Hou et al., 2022). Another study also discovered that SCFA production was reduced in mice on a low-fiber diet, which could suppress the immune system, particularly by reducing the activity and number of cytotoxic T cells required for anti-PD-1 therapy. This may lead to a worse response of tumors to anti-PD-1 treatment (Spencer et al., 2021). Furthermore, another study showed that butyrate inhibited the upregulation of CD80/CD86 on dendritic cells and the expression of Inducible T-cell Co-Stimulator on T cells in a mouse model, thereby impairing the recruitment of effector and memory T cells targeting tumor antigens. These changes may weaken the efficacy of anti-CTLA-4 therapy (Coutzac et al., 2020). Clinical studies also support these findings, suggesting that high levels of butyrate and propionate may enhance the function of immunosuppressive cells, such as Treg cells, and alter the immune microenvironment, thereby limiting the effectiveness of CTLA-4 inhibitors (Coutzac et al., 2020). The dual role of SCFAs on ICIs highlights the complexity of the effects of gut microbiota metabolites on the host, emphasizing the importance of continued investigation to determine the specific mechanisms.

Inosine, produced by specific commensal bacteria such as *Lactobacillus johnsonii*, has been shown to enhance the efficacy of ICB therapy. Immunotherapy-induced disruption of intestinal barrier function facilitates the systemic translocation of inosine. Inosine promotes T-helper 1 (Th1) differentiation by engaging the adenosine A2A receptor on T cells, thereby facilitating the recruitment of IFN-γ–producing CD4^+^ and CD8^+^ T cells within the tumor microenvironment, which augments the antitumor response induced by both anti-PD-1 and anti-CTLA-4 therapies (Liu et al., 2023). Notably, the immunomodulatory effect of inosine is context-dependent and may require a pro-inflammatory tumor microenvironment to exert its full therapeutic potential.

In a recent study, supplementation with *Lactobacillus johnsonii* or its microbial metabolite IPA also significantly improved the therapeutic outcome of ICB therapy in mice (Jia et al., 2024). The study demonstrated that *Lactobacillus johnsonii* collaborates with *Clostridium sporogenes* to produce IPAs, which then promote the driness of CD8 + T cells by increasing H3K27 acetylation in the T cell factor 7(Tcf7) superenhancer region. This epigenetic modification regulates the memory and persistence of T cells to support a more robust and sustained immune response, thereby improving the effectiveness of immunotherapy. Therefore, IPA supplementation promotes the expansion of CD8 + T cells, a key population of anti-tumor immune response, which helps to improve the cytotoxic ability of T cells against tumor cells. Based on this, the study found that *L. johnii* and its metabolite IPA can improve ICB reactivity and significantly improve the therapeutic efficacy of immune checkpoint inhibitors such as anti-PD-1 antibodies (Jia et al., 2024).

Another gut microbiome derived metabolite, gallic acid (GA), produced by microbial metabolism of dietary polyphenols such as tannins, has been found to enhance the efficacy of ICIs (Deng et al., 2022). GA acts by interfering with the phosphorylation process of signal transduction and activator of transcription 3 (STAT3). In this way, it leads to decreased expression of the ubiquitin-specific peptidase 21 (Usp21) gene. The subsequent reduction in Usp21 gene expression creates a chain reaction that results in decreased stability of FOXP3 and PD-L1 proteins. At the same time, FOXP3 and PD-L1 are closely related to the immunosuppressive function of Treg cells, and the weakening of their stability will cause Treg cells to reprogram to show a phenotype more similar to Th1, reducing their inhibitory activity. The researchers conducted further validation using a CRC mouse model, in which significant changes were observed when GA was combined with an PD-1 antibody. The expression levels of FOXP3 and PD-L1 in tumor infiltrating Treg cells were significantly decreased. At the same time, the production of IFN-γ in CD8 + T cells increases. In summary, GA can effectively improve the therapeutic effect of anti-PD-1 and anti-PD-L1 antibodies.

Studies have shown that the exopolysaccharide EPS-R1, produced by *Lactobacillus delbrueckii* subsp. *Bulgaricus* OLL1073R-1, can stimulate the production of CCR6^+^ CD8^+^ T cells in both mice and humans (Kawanabe-Matsuda et al., 2022). Oral administration of EPS-R1 in mice increases the number of CCR6^+^ CD8^+^ T cells in Peyer’s patches. These cells are capable of migrating to CCL20-expressing tumor tissues, including CRC, and exhibit enhanced IFN-γ production, thereby improving the tumor microenvironment (Nandi et al., 2021). This immunomodulatory effect of EPS-R1 significantly enhances the efficacy of ICIs, including anti-PD-1, anti-PD-L1, and anti-CTLA-4 antibodies, in treating CCL20-expressing colorectal tumors. These findings suggest that dietary intake of Lactobacillus-derived products containing EPS-R1 may represent a promising strategy to modulate specific T cell subsets and optimize the tumor microenvironment, ultimately improving the outcomes of immunotherapy for CRC.

Building upon the contribution of microbial metabolites to the improvement of immunotherapeutic outcomes, another study demonstrated that Limosilactobacillus reuteri (LR) produces indole-3-aldehyde (I3A), a tryptophan-derived metabolite that activates the AHR, thereby promoting IFN-γ and enhancing anti-tumor immune responses. Based on this finding, researchers developed a nanotechnology-engineered LR strain (LR-S-CD/CpG@LNP), which is loaded with an immunoadjuvant (CpG) and carbon dots (CDs), endowing it with photothermal and photodynamic properties. Experimental results showed that oral administration of LR-S-CD/CpG@LNP in mice modulated gut microbial metabolism and enhanced the tryptophan metabolic pathway, leading to increased I3A production (Xu et al., 2025). This, in turn, improved intestinal immune activity and markedly boosted the therapeutic effectiveness of immunotherapy against CRC.

The potential therapeutic impact of gut microbial metabolites extends beyond the prevention of immunosuppression; it also encompasses the mitigation of immune-related adverse events (irAEs). Dysbiosis of the gut microbiota may lead to immune dysregulation and an increased risk of irAEs. Studies have shown that microbial imbalance is associated with the exacerbation of irAEs, such as colitis, dermatologic reactions, and hepatotoxicity—common side effects observed in patients undergoing ICI therapy (Tan et al., 2022). Bacteria with anti-inflammatory properties, such as Bifidobacterium, and their metabolites may help alleviate immune-related inflammation. Restoring a healthier microbial balance could potentially mitigate the intensity of these immune-related complications, consequently enhancing both the tolerability and effectiveness of cancer immunotherapy. However, further clinical and mechanistic studies are needed to elucidate the specific relationships between gut microbial metabolites and irAEs, providing a foundation for future therapeutic strategies.

### 4.4 Fecal microbiota transplantation

Fecal microbiota transplantation (FMT) is an emerging microbiota-centered intervention that that entails transferring a diverse and stable community of gut microbiota from the feces of a healthy donor into the gastrointestinal tract of a recipient (Kaźmierczak-Siedlecka et al., 2020). Initially developed for the treatment of recurrent Clostridioides difficile infection, FMT has gained increasing attention in recent years due to its potential in correcting gut microbiota dysbiosis in various diseases, including CRC (Baunwall et al., 2020). As a novel microecological intervention, FMT reconstructs a healthy gut microbiota and effectively modulates the metabolic landscape within the tumor microenvironment. In one study using a CRC mouse model, FMT significantly improved survival, with approximately 90% of mice in the FMT-treated group surviving, compared to only about 50% in the untreated group (Yu et al., 2023). FMT was shown to restore heterogeneity and richness in gut microbiota of CRC mouse models, notably increasing the abundance of beneficial bacteria and reducing the proportion of harmful taxa. Furthermore, FMT alleviated intestinal inflammation by upregulating the anti-inflammatory cytokine IL-10 and reduced the accumulation of immunosuppressive Foxp3^+^ Tregs, thereby enhancing anti-tumor immune responses and suppressing CRC progression. In another study, researchers also applied FMT to CRC mouse models and found that FMT could restore both microbial composition and metabolic profiles, suggesting its therapeutic potential in CRC (Song et al., 2024). FMT significantly influenced the composition of microbial metabolites by increasing the production of beneficial SCFAs, particularly butyrate, while reducing the accumulation of carcinogenic SBAs. Moreover, FMT has been reported to enhance tumor sensitivity to conventional therapies such as chemotherapy and immunotherapy by reshaping the gut microbiota (Kang and Cai, 2021). As a promising adjunctive or standalone strategy, FMT offers a feasible and innovative approach to microbiota-targeted therapy in the management of CRC.

### 4.5 Phage therapy

The use of bacteriophages to target specific pathogenic bacteria and modulate their metabolic products has emerged as a potential therapeutic strategy in CRC management (Han et al., 2025). Studies have shown that phage therapy can selectively reduce the abundance of harmful bacteria and thus indirectly alter the composition of gut microbial metabolites. This modulation can influence host immune responses and inflammatory status, ultimately having a beneficial impact on the treatment of CRC. In a rather representative study, researchers loaded dextran based nanoparticles loaded with the chemotherapeutic drug irinotecan and combined them with engineered phages targeting the tumor-promoting bacterium Fn in experiments using phage-guided nanotechnology to modulate the gut microbiota in mouse models of CRC (Kannen et al., 2019). The experimental results are exciting, and these phage-guided nanoparticles not only cleverly promote the growth of beneficial *Clostridium butyricum* production, inject positive factors into the intestinal microecological environment, but also substantially enhance the anti-tumor effect of irinotecan and significantly improve the effectiveness of treatment. This example of research strongly demonstrates that phage therapy holds great potential in modulating the gut microbiota and its metabolites. Although bacteriophages have shown promising prospects in the field of modulating the gut microbiota and its metabolites, it is undeniable that their use in CRC therapy is still in its infancy (Liping et al., 2024). In the practical application process, there are still many urgent challenges to be solved. These include poor *in vivo* stability, limited targeting specificity, potential immunogenicity, and the lack of safe and effective delivery methods (Cui L. et al., 2024). Addressing these issues is essential for advancing phage-based interventions.

## 5 Conclusion and prospect

Gut microbiota metabolites are now recognized as key mediators in the pathogenesis of CRC, acting as biochemical messengers that link microbial dysbiosis to tumor initiation and progression. These small molecules span a wide range of types and collectively influence essential processes in the colorectal tumor microenvironment, including chronic inflammation, immune surveillance, epithelial cell proliferation, and apoptosis. Some metabolites exert tumor-suppressive effects, for instance, SCFAs help maintain intestinal barrier integrity and induce cancer cell apoptosis, while others promote carcinogenesis through mechanisms such as inducing genomic instability or modulating host cell signaling in favor of tumor progression. Therefore, the overall impact of the gut metabolic network on CRC depends on a delicate balance of these opposing effects, which can vary depending on factors such as dosage and context (Zhang W. et al., 2021).

Recent studies have highlighted the potential of microbial metabolites as diagnostic or prognostic biomarkers for CRC (Li et al., 2022). Since a majority of metabolites in feces and urine originate from the microbiota, metabolomic analysis offers a non-invasive window to monitor tumor-associated microbial activity. Integrative analyses combining metagenomics and metabolomics have identified several metabolite panels with high diagnostic accuracy. In addition to early detection, metabolite biomarkers also hold promise in prognostication, as well as in monitoring treatment responses and disease relapse. However, further validation in large cohorts and standardization of detection methods are needed to ultimately translate these findings into clinical practice. In terms of treatment, gut microbiota metabolites are being actively explored as targets and tools for CRC therapy. In addition to dietary modulation to alter the production of microbial metabolites, the combination of beneficial metabolites or microbiota modulators with chemotherapy or immunotherapy has also shown promising results. Furthermore, FMT to introduce beneficial microbiota and bacteriophage therapy targeting CRC-associated pathogens provide additional therapeutic options for CRC. Although these methods are still in the experimental stage, microbiome-based therapies are opening new dimensions in CRC treatment. By focusing on the tumor-supportive microbial ecosystem rather than just the cancer cells themselves, these innovative approaches hold the potential to enhance the effectiveness of CRC therapies. Despite strong evidence linking gut metabolites to the pathogenesis of CRC, several important limitations still impact our current understanding. First, findings across different studies are not always consistent. Variations in study design, sample types, and analytical techniques can lead to discrepancies in the metabolites identified as significant (Peng et al., 2021). It is increasingly clear that the lack of standardized protocols for sample collection, processing, and analysis hinders the reproducibility of results. Fecal, blood, or tissue samples may produce different metabolite profiles, and the outcomes can vary depending on the techniques used, such as gas chromatography–mass spectrometry or nuclear magnetic resonance. Therefore, there is an urgent need for larger cohorts and standardized sample preparation and metabolomic analysis methods to ensure the robustness of the identified biomarkers and mechanisms. Source attribution is another challenge. Many metabolites of interest, such as certain amino acid derivatives or vitamins, can be produced both by the microbiota and the host, or derived from the diet. Untangling the source of a metabolite detected in patients—whether microbial, host, or dietary—can often be challenging. Moreover, current research tends to focus on a relatively small subset of metabolites, leaving many compounds underexplored. The human gut metabolome contains thousands of molecules; however, most studies have concentrated on short-chain fatty acids, a few bile acids, and well-known toxins such as colibactin or H₂S. Many other bacterial products’ roles in CRC remain insufficiently studied. It is likely that within these metabolites lies crucial biological insight into cancer biology. Similarly, the gut mycobiome and virome have been relatively neglected. These interact with bacterial communities and hosts, producing metabolites that influence bacterial metabolism (El Haddad et al., 2022). Addressing these issues will require expanding our research perspective to consider the gut microbiota as a complex and diverse ecosystem, where each component may impact the development of CRC.

Looking ahead, integrating gut microbial metabolites into CRC management will require interdisciplinary and innovative research efforts. Incorporating host genomics, diet, and lifestyle factors into microbiome studies is crucial. The interaction between the host genome and microbiota may help explain why certain individuals are more sensitive to the pro-carcinogenic effects of microbial metabolites, thereby opening new avenues for personalized strategies aligned with an individual’s specific microbiome and metabolic characteristics. In terms of treatment, precision microbiome manipulation holds great promise. While traditional dietary and probiotic interventions remain relatively crude, engineered microbiota-based therapies are expected to emerge in the coming years. Synthetic biology enables bacteria to be genetically programmed to perform desired functions in the gut. In CRC, researchers can design probiotics to secrete anti-cancer compounds, neutralize carcinogenic metabolites, or deliver immune-modulating signals in the tumor microenvironment. These “designer probiotics” can be tailored to individual microbiomes, enhancing therapeutic specificity (Siguenza et al., 2024). Similarly, phage therapy can be bioengineered to improve efficacy and safety, targeting harmful bacteria and delivering gene-editing tools to knock out genes responsible for toxic metabolites. Additionally, developing small-molecule inhibitors to block microbial enzymes represents an attractive strategy for chemically modulating microbiome metabolism without eradicating microbes. In conclusion, gut microbial metabolites are critically involved in CRC by contributing to tumor development, serving as indicators of disease onset and progression, and offering novel therapeutic opportunities. Despite the challenges in decoding these complex biochemical interactions, the field is rapidly advancing. Continued interdisciplinary efforts and well-designed clinical trials will help uncover deeper insights into the relationship between CRC and gut microbial metabolites, offering new opportunities for more effective treatment strategies.
